# Practical solutions to resolve social barriers to hepatitis C treatment initiation among people who inject drugs: a qualitative study

**DOI:** 10.1186/s12954-024-01136-1

**Published:** 2024-12-20

**Authors:** Phyo Aung, Margaret Hellard, Paul Dietze, Bek Petrovic, Peter Higgs, Mark Stoové

**Affiliations:** 1 Disease Elimination Program, Burnet Institute, 85 Commercial Road, Melbourne, VIC 3004 Australia; 2 Department of Epidemiology and Preventive Medicine, Monash University, 553 St Kilda Road, Melbourne, VIC 3004 Australia; 3 Department of Infectious Diseases, Alfred Health & Monash University, 85 Commercial Road, Melbourne, VIC 3004 Australia; 4 National Drug Research Institute and Enable Institute, Curtin University, 85 Commercial Road, Melbourne, VIC 3004 Australia; 5 Department of Public Health, La Trobe University, HS 2, Room 519, Bundoora, VIC 3083 Australia

**Keywords:** Practical solutions, Hepatitis C treatment, Behaviour change, People who inject drugs, Motivators

## Abstract

**Background:**

We aimed to identify motivators for people who inject drugs to pursue treatment for hepatitis C virus (HCV) infection and uncover opportunities that could make treatment more appealing.

**Methods:**

Between November 2023 and January 2024, we conducted semi-structured interviews with 15 HCV RNA-positive individuals with a history of injecting drug use and self-reported as either untreated or treated but delayed treatment for more than 6 months. Thematic and framework data analysis was employed and interpreted using the Capability, Opportunity and Motivation (COM-B) framework of behaviour change.

**Results:**

The findings suggest that a combination of stability through secure housing, mental readiness and overcoming drug dependence supported by OAT (Capability), accessible and convenient healthcare like mobile outreach services coupled with financial incentives (Opportunity), and supportive relationships (Motivation) could serve to help people who inject drugs take up HCV treatment.

**Conclusion:**

Alcohol and other drug and primary care services for people who use drugs should adopt person-centred approaches and recognise the gradual nature of behavioural change to foster empathy and supportive relationships to promote engagement in HCV care. Additionally, integrating HCV treatment with mobile outreach services and providing practical assistance, such as housing and financial incentives, are critical to ensuring that individuals remain engaged in HCV care.

## Background

In 2015, the World Health Organization (WHO) established global targets to eliminate hepatitis C virus (HCV) infection as a public health threat by 2030 [1]. These targets included treating 80% of people with HCV, reducing new HCV infections by 90% and liver-related mortality by 65% [1]. In 2016, Australia listed direct-acting antivirals (DAAs) on the pharmaceutical benefits scheme, thereby ensuring universal access to these medicines [2]. With the availability of DAAs, the estimated number of people living with chronic HCV decreased from 188,690 at the end of 2015 to 74,400 by the end of 2022 [3]. Early DAA treatment rates appeared to put Australia on track to achieving HCV elimination [4]. However, an estimated 40% of individuals living with HCV remain untreated and treatment uptake, which peaked in 2016, has declined steadily [3]. Australia now appears unlikely to achieve the elimination targets by 2030 if annual treatment numbers stay at their current levels [5].

In Australia, HCV is concentrated among people who inject drugs [3, 6, 7]. Interrupting the risk of onward transmission of HCV in this group through treatment and cure (treatment as prevention) is key to achieving HCV elimination [8–10]. The simplicity of DAA treatment and its minimal side effect profile compared to the previously available interferon-based therapies have enabled DAA treatment in primary care settings, including specialist alcohol and other drug (AOD) treatment services. This has led to higher treatment acceptability and uptake among people who inject drugs [3, 11, 12]. New point-of-care testing technologies have also facilitated simplified testing and treatment models, peer-based approaches to support engagement in care [6], and higher treatment acceptability and uptake among people who inject drugs.

Despite these advantages of DAAs, barriers to initiating HCV treatment among people who inject drugs persist. Previous studies have identified various individual and structural barriers and facilitators to HCV testing and treatment uptake amongst people who inject drugs [13–24]. The mostly asymptomatic nature of chronic HCV infection has been found to limit the perceived urgency of seeking HCV care [25]. In addition, multiple competing health and social needs, such as family and childcare responsibilities, transport, employment, unstable housing, drug use and other health conditions, including mental health, have been found to take precedence over accessing HCV treatment [14, 25–28]. To address these challenges, financial incentives or reimbursements have been considered a potential strategy to alleviate these competing priorities, with at least one study [29] underway to assess the effectiveness of financial support in motivating people to initiate HCV treatment. Furthermore, opioid agonist therapy (OAT) has been shown to improve HCV treatment initiation [30, 31]. However, health service attributes such as accessibility and stigma continue to deter treatment uptake [14, 32].

Our earlier research identified that competing priorities in life and health service attributes, including stigma, were the persistent barriers to treatment initiation among people who inject drugs [26]. It is, however, important to identify strategies that can promote behaviour change and subsequent HCV treatment uptake. The COM-B model by Michie et al. [33] proposes three key factors (Capability, Opportunity, Motivation) are necessary for behaviour change (Behaviour). The model suggests that for a behaviour (B) to occur, individuals must be physically and psychologically capable (C) and have the social and physical opportunity (O) to do the behaviour, be more motivated (M) to do that behaviour than any other competing actions. Motivation includes basic drives, habits, impulses, and reflective processes like intentions and choices. The COM-B model provides a framework to explore how capability, opportunity, and motivation influence HCV treatment initiation among people who inject drugs.

While there is evidence on the barriers to HCV treatment among people who inject drugs, the literature provides limited guidance on strategies for behaviour change, with previous studies focusing mostly on barriers to treatment. Guided by the COM-B framework, this study aims to identify motivational contexts and actionable solutions to address the persisting barriers to HCV treatment initiation among people who inject drugs.

## Methods

### Design

We conducted semi-structured in-depth interviews about barriers to HCV treatment and practical strategies to reduce them and facilitate HCV treatment initiation. Participants were recruited from the Melbourne Injecting Dug User Cohort Study (SuperMIX) cohort. SuperMIX is a longitudinal cohort study designed to measure injecting trends, incidence of injecting cessation, and health service utilisation among people who inject drugs in Melbourne and Victoria. SuperMIX has been described in detail elsewhere [34].

### Recruitment

Participants were recruited using both purposive and convenience sampling. Eligible people were aged 18 years or older; currently enrolled in SuperMIX; had a positive HCV RNA test at their most recent SuperMIX interview; and self-reported never being treated for HCV or receiving no treatment for 6 months or more after their most recent SuperMIX HCV RNA positive result.

Participants who met the eligibility criteria were identified either by review of SuperMIX data in the 24 months preceding recruitment or at their most recent follow-up interview. A total of 53 individuals were identified as potentially eligible, however those without current available phone numbers were excluded. Further, some participants had yet to be informed of their most recent HCV test results, and so they too were excluded, leaving, 26 eligible individuals. A target sample of 20 participants was set initially, but recruitment ceased when data saturation was determined to have been reached, based on the overall quality and completeness of the data and the lack of emergence of novel themes across latter interviews.

Eligible participants who consented were interviewed by the authors (PH and/or PA) either via phone or face-to-face at convenient locations for the participants, including community health centres, a mobile van, Burnet Institute meeting rooms, cafes and quiet outdoor spaces. Study participants were reimbursed AUD40 for their time and expertise at the completion of the interview.

### Data collection

Interviews occurred between 17 November 2023 and 24 January 2024. The interview guide was informed by previous literature [14, 25, 26, 32, 35, 36] and discussion among co-authors.

The interviews focussed on potential motivators to the persisting barriers including stigma, social support, transportation and service access; health conditions and comorbidities, including mental illness; financial support and/or unemployment; and health literacy and knowledge about HCV treatment.

Interviews lasted 12–77 min (median:26 min), and were audio recorded using a personal handheld digital voice recorder.

Demographic details (age, gender, HCV RNA status, employment status, education status, and HCV treatment history) were sourced from SuperMIX.

### Data analysis

Interviews were transcribed verbatim by a professional transcription service. Participants were given pseudonyms to protect their identities. Transcripts were checked for accuracy, interviewer consistency and de-identification. Transcripts and interviewer notes were managed and organized using NVivo version 1.7 (2022). Themes were developed iteratively through a process of initial inductive coding; wherein raw data were analysed systematically to identify patterns and categories. Following this initial coding phase, the COM-B model was used as a deductive framework for data analysis. The co-authors conducted debriefing sessions and discussed the emerging themes and insights arising from the analysis to minimise their own biases and assumptions.

## Results

Fifteen participants (11 men, 4 women), aged 37–57 years, were interviewed. Eleven (73%) had completed year 10 of high school. Only two participants reported current employment and five reported unstable housing. All had a history of incarceration.

Based on their infection and treatment history, participants were categorised into two groups: untreated or treated but delayed, with delays defined as more than 6 months from diagnosis to treatment. Ten participants were untreated, with a median time from positive result to interview of 1.2 years (range 0.7 to 6.14 years). Five participants delayed treatment, with a median time to treatment of 3.45 years (range 0.6 to 5.17 years).

Using the COM-B model, findings were grouped into those relating to the themes of capability, opportunity and motivation.

### Capability

Many participants lacked knowledge about HCV transmission, consequences, and treatment options as well as skills and resources to navigate the health system. Rob (aged 46) who remained untreated, noted that this lack of awareness has prevented him from seeking treatment.

I don’t know which medication I’m supposed to be getting…I don’t know how often you need to pick up the pills… and where I can get treatment….

On the other hand, participants talked about how stability, described as secure housing, stable or inactive drug use and mental readiness, could enhance the capability needed for HCV treatment. Reese (aged 46) described that housing stability not only affected access to treatment but was essential for successful completion of treatment, highlighting the challenge of maintaining consistency in treatment when experiencing housing instability.

You got to be stable. You can’t be jumping around different places or else you are never going to take it [hep C tablets] like you should.

Participants also highlighted the importance of a holistic person-centred approach to healthcare, including the integration of housing support with HCV treatment. Mike (aged 46) explained how support from social services to obtain stable housing, such as organising paperwork, enabled him to initiate HCV treatment.

… I changed because I got housing… He [health worker from a community drug and alcohol clinic] helped me arrange to get my place. He put all the paperwork together. He’s the one who helped me out … Having the right person and understand you, and getting the right help has helped me [change and take up hep C treatment].

The participant described how mental readiness to overcome drug dependence and a commitment to a stable life, including overall health, were crucial prerequisites for starting HCV treatment. Phil (aged 46) who was considering OAT explained the interconnectedness of mental preparedness and life stability, including freedom from drug dependence, and the preferences for addressing all health issues simultaneously.

You have to give up drugs… Getting suboxone shot means no more heroin. You need to be mentally ready… which means stability in your life…pay my rent and utilities. I want to be with my kids…When I am ready, I want to do it all at once [ HCV treatment and OAT]. It makes sense to do it all once.

Participants emphasised the crucial role of social and mental health support in motivating and facilitating their journey through HCV treatment. Many participants appeared to lack adequate social or counselling support and frequently expressed the need for “someone to talk to”, especially when struggling with mental illness. Jenny (aged 40, group 2) explained that mental health support might have assisted them with faster treatment uptake.

If I had someone encouraging during that time [while struggling with mental illness] or take me there [GP clinic], I would have taken the treatment [DAA] faster.

### Opportunity

Missed opportunities for HCV treatment often arise from healthcare providers not prioritising the condition and the presence of stigma within health services. This lack of prioritisation may have stemmed from time limitations and competing care priorities, such as treatment of substance use. As Jenny (aged 40) described:

I mentioned it to the doctor [from a community AOD clinic] a few times and those couple of visits were to fix up my methadone script. I said, “I’ve been told I’ve got hep C for a while now; can we hurry up and do the medicine?” … But that was my fault too because like I was late to some appointments and ran out of time.

Many participants, particularly those living outside metropolitan areas, mentioned that structural barriers related to geographical distance, discouraged them from seeking treatment, highlighting a missed opportunity for treatment. Aaron (aged 48) who remained untreated, suggested that assistance with transport would lower this barrier:

One time, I’d be in a regional area [where he lives], then I’d be in the city. The regional area has appointments scheduled for 10:00 in the morning, so it’d be hard to get there … Arranging transport, money for a ticket, to get to and from the appointments would be helpful to get onto [hepatitis C] treatment.

Participants mentioned that competing priorities such as caregiver responsibilities, complicated their ability to seek treatment. Ricki (aged 37) who was untreated for reinfection, described how these challenges made it difficult to prioritise their health.

I have a whole list of things to do. My mum broke her leg, and I am looking after her. I’ve just moved into the flat and I’ve to put together all the furniture and stuff. Once everything’s finalised, I can get treatment for Hep C.

However, Ricki had previously received HCV treatment through a study that offered financial incentives. Ricki explained that the incentives provided an opportunity to seek treatment.

You guys [Burnet research team] called me up and it [ the study] was there. There’s also money incentive, which was always a yes for me. Like offer a clear incentive and more likely to go towards.

In addition to financial incentives, many participants acknowledged the crucial role that the mobile outreach services such as the Burnet research van played in providing HCV treatment by offering accessible and immediate health services. Bianca (aged 39) who had HCV testing and was about to commence the treatment, explained that the van’s accessibility, convenience, and community engagement provided the opportunity for them to receive HCV-related medical support that they had not previously sought out.

It wasn’t until about, about maybe four weeks ago, I came across a girl who is a nurse at the Hospital. She was in one of your vans, and she did my bloods and my vitals. She’s amazing…It was only because I happened to walk upon your van, you’re able to support me. No one’s had the chance to support me because I have not asked for it.

Mike (aged 46) also talked about the importance of sustaining the quality of outreach services, building trust and ensuring service continuity in providing ongoing opportunities for HCV treatment.

What could have been helpful would be more people getting out and seeing what people need to help there. That seems to change a bit. There was a lot of help before. When I was on the streets, missions are coming in to give clothes and stuff like that … I found out about this service [primary care health service for people using drugs]. They are the only ones who helped me. I became healthier. The workers have changed now. It was a great team before COVID. Now, they are all new workers, they are all young and won’t have nothing to do with them.

Importantly, OAT was identified as a crucial opportunity for HCV treatment. Participants emphasised the value of flexible treatment options; injectable buprenorphine was viewed as more convenient and effective than daily oral methadone. Flexible treatment options were seen as integrated solutions that address both substance use and HCV infection simultaneously. Reese (aged 46, group 1) explained:

I want to convert from methadone to the injection [long-acting buprenorphine], so I wanted to do that first and at the same time start the treatment [hep C treatment] …. It’d be better just to get it all done at the same time.

### Motivation

Stigma can significantly demotivate individuals from seeking HCV treatment. Participants reflected how stigma experienced through interactions with their service providers, including judgmental language and biased assumptions about their lifestyle led to a loss of motivation to treatment. Despite his previous willingness to undergo treatment, Shane (aged 48) hesitated to seek treatment for his reinfection due to negative experiences with his new doctor. Shane disclosed:

I don’t want her [current doctor] to do it [ Hep C treatment], because, I’ve had troubles with her. She’s just judgemental. Like, “an ex-druggie or still a druggie,”. I just get treated different because of the way I look and because I got me long hair. I’m finding that hard.

On the other hand, supportive relationships with clinicians can motivate health seeking behaviour. Participants with treatment histories reflected strong relationships with service providers facilitated past HCV treatment by reducing stigma associated with drug use and HCV infection. Shane attributed his previous decision to undergo treatment to his rapport with his former doctor who had experience in AOD treatment.

It’s having a good doctor that understands you. It was just nice, having somebody be with you and didn’t treat you like a number.

The combination of stigma and providers’ apathy towards HCV treatment can leave individuals feeling unsupported and delay treatment initiation; in contrast, patient-centred care can provide reassurance and instil confidence. Rob (aged 46), who was preparing to receive treatment, articulated how contrasting experiences with clinicians led to different outcomes for his HCV treatment.

With the first doctor [local GP], he did not take it [my positive HCV test result] seriously. He wants me to get another blood test. He didn’t even ask me if I ‘ve repeated the blood test. I can see the demeanour in the person. He doesn’t care … I went back to the old clinic [primary care health service for people injecting drugs], the doctor there took it more seriously. He took the blood again but said that hep C can go away by itself and he’s just doing it for precaution … He’s a great guy.

Participants also described the involvement of peers, families, and friends as crucial in facilitating the initiation of HCV treatment, supporting healthcare engagement through nudges, alleviating fears, or demystifying misconceptions around side effects. Furthermore, the desire to do better for their families or children can be a powerful motivator for individuals to prioritise their health and initiate treatment. Joe (aged 47), who successfully completed treatment after an initial delay, highlighted the value of his friends and peers in providing reassurance and easing his concerns about side effects.

I was worried about side effects. That was really my only concern. But I spoke to a lot of other people [friends and peer networks] who had done it, and they were fine with it … And my mother and my kids were there for me and motivated me.

## Discussion

Our research identified potential motivators to HCV treatment initiation using a structured approach based on the COM-B model. Our findings indicate that a combination of stability through secure housing, mental readiness and overcoming drug dependence supported by OAT (Capability), accessible and convenient healthcare like mobile outreach services coupled with financial incentives (Opportunity), and supportive relationships (Motivation) may work to improve HCV treatment uptake among people who inject drugs, the key population needed to reach for HCV elimination.

Our findings indicate that ‘stability’ improves the capacity for HCV treatment, with stability generally described as being free from drug dependence, maintaining mental well-being, and having secure housing. Change towards stability involves a combination of practical support such as housing as well as biomedical interventions such as OAT and comprehensive care. This process was described as gradual, requiring continuous personal growth, and empathetic support from healthcare providers. Treloar et al. [37] underscored the concept of “structural competency”, highlighting the importance for healthcare providers and policymakers to recognise how structural issues disproportionately affect the choices and health behaviours of people who inject drugs. They emphasised the need for person-centred approaches to overcome these barriers and improve service engagement.

Our findings show that OAT plays a crucial role in both improving capability and creating opportunities for HCV treatment. By stabilising drug use, OAT enables individuals to better concentrate on their overall health, including HCV treatment. Participants expressed the view that traditional options like methadone were considered less convenient than long-acting injectable buprenorphine due to the need for frequent dosing at a pharmacy. Research suggests that novel long-acting OAT presents several advantages, including reduced stigma at pharmacies/clinics and increased flexibility for travel and work [38, 39] Nonetheless, insufficient contact with healthcare services may disrupt clinical support systems, including opportunities for HCV treatment [40]. Studies have suggested that integrated pharmacy HCV care models that provide access to care at OAT dispensing may overcome barriers associated with less frequent presentation to long-acting OAT prescribers [39].

People who inject drugs face a myriad of socioeconomic challenges such as the financial burden associated with transportation, and other competing priorities, all of which can deter HCV treatment seeking. The traditional medical model alone is unlikely to address these challenges effectively and innovative strategies are needed. Consistent with previous research [41–44], our study signalled that financial reimbursements can not only incentivise treatment uptake but offset medical expenses and compensate for the time invested in seeking HCV treatment. Our study also highlights the participants’ acceptance and appreciation of our research van, and the role of mobile outreach services in HCV care. Mobile outreach services can help eliminate some of the structural barriers by bringing care directly to patients, making the service both flexible and convenient. Both locally and internationally, successful examples of such outreach services have been demonstrated, including a similar initiative that involves a mobile van providing testing and treatment in Queensland, Australia [45]. These services often adopt a personalised approach, which can help overcome stigma-inducing environments.

Our study found that supportive relationships present within tailored AOD services or community health services are critical in creating a conducive and welcoming environment that encourages individuals to pursue treatment [13, 14]. A non-stigmatising approach not only promotes continued engagement with services but enhances awareness of the implications of untreated HCV infection and the availability of treatment options [14, 25, 46]. However, even within these targeted health services, prioritisation of HCV treatment varies with time constraints and the complexity of medical and social conditions associated with drug use. For example, recent analysis of data from a network of OAT providers in Australia showed very low levels of HCV testing in the 12 months following initiation of therapy [47], This finding highlights the need to strengthen integrated HCV care in AOD settings and enable primary care providers to hold longer consultations and educate patients about HCV treatment. “Slow medicine” [48], which prioritises comprehensive patient care over rushed interventions, is needed.

Our study has several limitations. Firstly, our small sample size may affect its generalisability by preventing full capture of the breadth of experiences of people affected by HCV. Secondly, recall biases may have influenced the data accuracy. Thirdly, whilst the interviews explored factors identified by participants as reasons for not seeking treatment, they may not have captured timing-related nuances that could impact treatment decisions. Fourthly, our study included a small number of women, and gender-specific experiences were not explored as these were not the study’s main objectives.

## Conclusions

To scale up HCV treatment, it is essential to support primary care services for people who use drugs through efforts such as stigma reduction, investing in longer consultations, and establishing supportive relationships. AOD and primary care services for people who inject drugs should be integrated with mobile outreach services to reduce social and structural barriers to HCV treatment and complemented by practical assistance, such as financial and housing support. Recognising that behavioural change for people who inject drugs is gradual and influenced by multiple structural issues, service providers must adopt patience, empathy, and understanding throughout the change process.

## Data Availability

The aggregated data that support the findings of this study are available from the corresponding author, PA, upon reasonable request. The data requests are subject to approval by the chief investigators and the working group.

## Abbreviations

AOD: Alcohol and other drugs
HCV: Hepatitis C virus
OAT: Opioid Agonist Therapy
WHO: World Health Organization

## References

[1] World Health Organization. Global Health Sector strategies on, respectively, HIV, viral hepatitis and sexually transmitted infections for the period 2022–2030. Geneva; 2022.

[2] Dore GJ. Elimination of hepatitis c in Australia by 2030: a decade and counting. Aust Prescr. 2021;44(2):36–7. 33911328 10.18773/austprescr.2021.003 PMC8075743

[3] Burnet Institute and Kirby Institute. Australia’s progress towards hepatitis C elimination: Annual Report 2023. Melbourne; 2023.

[4] Razavi H, Sanchez Gonzalez Y, Yuen C, Cornberg M. Global timing of hepatitis C virus elimination in high-income countries. Liver Int [Internet]. 2020;40(3):522–9. 10.1111/liv.14324 10.1111/liv.14324 31815353

[5] Kwon JA, Dore GJ, Hajarizadeh B, Alavi M, Valerio H, Grebely J et al. Australia could miss the WHO hepatitis C virus elimination targets due to declining treatment uptake and ongoing burden of advanced liver disease complications. PLoS One [Internet]. 2021;16(9):e0257369. 10.1371/journal.pone.0257369 10.1371/journal.pone.0257369 PMC8445464 34529711

[6] Alisa Pedrana S, Munari M, Stoove’ J, Doyle MH. The phases of hepatitis C elimination: achieving WHO elimination targets. Melbourne; 2021. 10.1016/S2468-1253(20)30366-6 33308435

[7] Iversen J, Dore GJ, Starr M, Catlett B, Cunningham P, Geddes L et al. Estimating the Consensus Hepatitis C Cascade of Care among people who inject drugs in Australia: pre and post availability of direct acting antiviral therapy. Int J Drug Policy. 2020;83. 10.1016/j.drugpo.2020.102837 32645585

[8] Rockstroh JK, Boesecke C. Hepatitis C Virus Treatment as Prevention: Challenges and Opportunities in Men Who Have Sex With Men. J Infect Dis [Internet]. 2020;222(Supplement_9):S782–8. 10.1093/infdis/jiaa096 10.1093/infdis/jiaa096 33245348

[9] Hellard M, Schroeder SE, Pedrana A, Doyle J, Aitken C. The elimination of hepatitis C as a public health threat. Cold Spring Harb Perspect Med. 2020;10(4). 10.1101/cshperspect.a036939 PMC7117951 31712223

[10] Hellard M, McBryde E, Davis RS, Rolls D, Higgs P, Aitken C, et al. Hepatitis C transmission and treatment as prevention – the role of the injecting network. IDJP. 2015;26(10):958–62. 10.1016/j.drugpo.2015.05.006 26072105

[11] Valerio H, Alavi M, Conway A, Silk D, Treloar C, Martinello M, et al. Declining prevalence of current HCV infection and increased treatment uptake among people who inject drugs: the ETHOS Engage study. Int J Drug Policy. 2022;105:103706. 35533635 10.1016/j.drugpo.2022.103706

[12] Richmond JA, Wallace J. Implementation of hepatitis C cure in Australia: one year on. J Virus Erad. 2018;4(2):115–7. 29682304 10.1016/S2055-6640(20)30254-5 PMC5892669

[13] Høj SB, Jacka B, Minoyan N, Artenie AA, Bruneau J. Conceptualising access in the direct-acting antiviral era: An integrated framework to inform research and practice in HCV care for people who inject drugs. Int J Drug Policy [Internet]. 2019;72:11–23. 10.1016/j.drugpo.2019.04.001 10.1016/j.drugpo.2019.04.001 31003825

[14] Heard E, Smirnov A, Massi L, Selvey LA. Personal, provider and system level barriers and enablers for hepatitis C treatment in the era of direct-acting antivirals: experiences of patients who inject drugs accessing treatment in general practice settings in Australia. J Subst Abuse Treat. 2021;127(November 2020). 10.1016/j.jsat.2021.108460 34134878

[15] Stagg HR, Surey J, Francis M, MacLellan J, Foster GR, Charlett A, et al. Improving engagement with healthcare in hepatitis C: a randomised controlled trial of a peer support intervention. BMC Med. 2019;17(1):1–9. 30929642 10.1186/s12916-019-1300-2 PMC6442435

[16] Valerio H, Alavi M, Silk D, Treloar C, Martinello M, Milat A, et al. Progress towards elimination of Hepatitis C infection among people who inject drugs in Australia: the ETHOS Engage Study. Clin Infect Dis. 2021;73(1):e69–78. 32421194 10.1093/cid/ciaa571 PMC8246809

[17] Bajis S, Grebely J, Cooper L, Smith J, Owen G, Chudleigh A, et al. Hepatitis C virus testing, liver disease assessment and direct-acting antiviral treatment uptake and outcomes in a service for people who are homeless in Sydney, Australia: the LiveRLife homelessness study. J Viral Hepat. 2019;26(8):969–79. 30980785 10.1111/jvh.13112

[18] Batchelder AW, Cockerham-Colas L, Peyser D, Reynoso SP, Soloway I, Litwin AH. Perceived benefits of the hepatitis C peer educators: a qualitative investigation. Harm Reduct J [Internet]. 2017;14(1):67. 10.1186/s12954-017-0192-8 10.1186/s12954-017-0192-8 PMC5622540 28962652

[19] Beiser ME, Smith K, Ingemi M, Mulligan E, Baggett TP. Hepatitis C treatment outcomes among homeless-experienced individuals at a community health centre in Boston. Int J Drug Policy. 2019;72:129–37. 30962036 10.1016/j.drugpo.2019.03.017

[20] Colledge S, Leung J, Larney S, Peacock A, Grebely J, Hickman M et al. Frequency of injecting among people who inject drugs: a systematic review and meta-analysis. Int J Drug Policy. 2020;76. 10.1016/j.drugpo.2019.102619 31864107

[21] Crowley D, Van Hout MC, Lambert JS, Kelly E, Murphy C, Cullen W. Barriers and facilitators to hepatitis C (HCV) screening and treatment - A description of prisoners’ perspective. Harm Reduct J. 2018;15(1):1–10. 30538000 10.1186/s12954-018-0269-z PMC6288965

[22] Richmond JA, Gallagher L, McDonald L, O’Sullivan M, Fitzsimmons C, Pedrana A. Achieving Hepatitis C elimination by using Person-Centered, nurse-led models of care: a discussion of Four International Case studies. Gastroenterol Nurs. 2020;43(4):303–9. 32665524 10.1097/SGA.0000000000000458

[23] Pourmarzi D, Smirnov A, Hall L, Thompson H, FitzGerald G, Rahman T. Enablers and barriers for the provision of community-based HCV treatment: a case study of a real-world practice. J Viral Hepat. 2020;27(5):484–96. 31958355 10.1111/jvh.13259

[24] Papaluca T, McDonald L, Craigie A, Gibson A, Desmond P, Wong D et al. Outcomes of treatment for hepatitis C in prisoners using a nurse-led, statewide model of care. J Hepatol [Internet]. 2019;70(5):839–46. 10.1016/j.jhep.2019.01.012 10.1016/j.jhep.2019.01.012 30654067

[25] Gunn J, McNally S, Ryan J, Layton C, Bryant M, Walker S, et al. Barriers to hepatitis C treatment among secondary needle and syringe program clients and opportunities to intervene. Int J Drug Policy. 2021;96:103387. 34330571 10.1016/j.drugpo.2021.103387

[26] Aung P, Goutzamanis S, Douglass C, Stoove M, Hellard M, Dietze P et al. Exploring opportunities for hepatitis C treatment uptake among people who inject drugs in Australia: a qualitative study. J Subst Use [Internet]. 2023;1–6. 10.1080/14659891.2023.2238317

[27] Crowley D, Cullen W, Lambert JS, Van Hout MC. Competing priorities and second chances - A qualitative exploration of prisoners’ journeys through the Hepatitis C continuum of care. PLoS One [Internet]. 2019;14(9):e0222186. 10.1371/journal.pone.0222186 10.1371/journal.pone.0222186 PMC6738615 31509571

[28] McColl R, Higgs P, Harney B. No, my name’s not on the lease at all: an interpretive phenomenological analysis of unstable housing and hepatitis C among people who inject drugs. Drugs, Habits Soc Policy [Internet]. 2024;ahead-of-p(ahead-of-print). 10.1108/DHS-08-2023-0034

[29] Fathima P, Jones M, D’Souza R, Totterdell J, Andric N, Abbott P, et al. Financial incentives to motivate treatment for hepatitis C with direct acting antivirals among Australian adults (the methodical evaluation and optimisation of targeted IncentiVes for accessing treatment of early-stage hepatitis C: MOTIVATE-C): protocol. Trials. 2024;25(1):387. 38886819 10.1186/s13063-024-08212-8 PMC11181591

[30] Gunn J, Higgs P. Directly observed hepatitis C treatment with opioid substitution therapy in community pharmacies: a qualitative study. Res Social Adm Pharm. 2020;16(9):1298–301. 31003763 10.1016/j.sapharm.2019.04.004

[31] Jones NR, Nielsen S, Farrell M, Ali R, Gill A, Larney S, et al. Retention of opioid agonist treatment prescribers across New South Wales, Australia, 2001–2018: implications for treatment systems and potential impact on client outcomes. Drug Alcohol Depend. 2021;219:108464. 33360851 10.1016/j.drugalcdep.2020.108464 PMC7855715

[32] Goutzamanis S, Doyle JS, Horyniak D, Higgs P, Hellard M. Peer to peer communication about hepatitis C treatment amongst people who inject drugs: a longitudinal qualitative study. Int J Drug Policy. 2021;87. 10.1016/j.drugpo.2020.102983 33126166

[33] Michie S, van Stralen MM, West R. The behaviour change wheel: A new method for characterising and designing behaviour change interventions. Implement Sci [Internet]. 2011;6(1):42. 10.1186/1748-5908-6-42 10.1186/1748-5908-6-42 PMC3096582 21513547

[34] Van Den Boom W, Quiroga M, del O’Keefe M, Kumar D, Hill D, Scott PL et al. N,. Cohort Profile: The Melbourne Injecting Drug User Cohort Study (SuperMIX). Int J Epidemiol [Internet]. 2021;dyab231. 10.1093/ije/dyab231 10.1093/ije/dyab231 34961882

[35] Skeer MR, Ladin K, Wilkins LE, Landy DM, Stopka TJ. Hep C’s like the common cold: understanding barriers along the HCV care continuum among young people who inject drugs. Drug Alcohol Depend. 2018;190:246–54. 30071457 10.1016/j.drugalcdep.2018.06.013 PMC6367928

[36] Paisi M, Crombag N, Burns L, Bogaerts A, Withers L, Bates L, et al. Barriers and facilitators to hepatitis C screening and treatment for people with lived experience of homelessness: a mixed-methods systematic review. Heal Expect Int J Public Particip Heal care Heal Policy. 2022;25(1):48–60. 10.1111/hex.13400 PMC8849376 34862710

[37] Treloar C, Schroeder S, Lafferty L, Marshall A, Drysdale K, Higgs P, et al. Structural competency in the post-prison period for people who inject drugs: a qualitative case study. Int J Drug Policy. 2021;95:103261. 33990057 10.1016/j.drugpo.2021.103261

[38] Barnett A, Savic M, Lintzeris N, Bathish R, Arunogiri S, Dunlop AJ et al. Tracing the affordances of long-acting injectable depot buprenorphine: A qualitative study of patients’ experiences in Australia. Drug Alcohol Depend [Internet]. 2021;227:108959. https://www.sciencedirect.com/science/article/pii/S0376871621004543 10.1016/j.drugalcdep.2021.108959 34450472

[39] Byrne CJ, Radley A, Inglis SK, Beer L, Palmer N, Duc Pham M et al. Reaching people receiving opioid agonist therapy at community pharmacies with hepatitis C virus: an international randomised controlled trial. Aliment Pharmacol Ther [Internet]. 2022;55(12):1512–23. 10.1111/apt.16953 10.1111/apt.16953 35538396

[40] Soyka M, Franke AG. Recent advances in the treatment of opioid use disorders-focus on long-acting buprenorphine formulations. World J Psychiatry. 2021;11(9):543–52. 34631459 10.5498/wjp.v11.i9.543 PMC8474991

[41] University of Sydney. The MotivateC Project [Internet]. 2024 [cited 2024 Jun 13]. https://motivatec-project.sydney.edu.au/about/

[42] Wohl DA, Allmon AG, Evon D, Hurt C, Reifeis SA, Thirumurthy H et al. Financial Incentives for Adherence to Hepatitis C Virus Clinical Care and Treatment: A Randomized Trial of Two Strategies. Open Forum Infect Dis [Internet]. 2017;4(2):ofx095. 10.1093/ofid/ofx095 10.1093/ofid/ofx095 PMC5499638 28695144

[43] Pedrana A, Munari S, Stoove M, Doyle JHM. Comment the phases of hepatitis C elimination: achieving WHO elimination targets. Lancet Gastroenterol Hepatol. 2021;6(1):6–8. 33308435 10.1016/S2468-1253(20)30366-6

[44] Palmer AY, Chan K, Gold J, Layton C, Elsum I, Hellard M et al. A modelling analysis of financial incentives for hepatitis C testing and treatment uptake delivered through a community-based testing campaign. J Viral Hepat [Internet]. 2021;28(11):1624–34. 10.1111/jvh.13596 10.1111/jvh.13596 34415639

[45] Conway B, Sharma S, Yung R, Yi S, Toniato G. Current Models to Address Obstacles to HCV Elimination. In: Yang L, Qi X, editors. Rijeka: IntechOpen; 2023. p. Ch. 5. 10.5772/intechopen.1001867

[46] Treloar C, Rance J, Grebely J, Dore GJ. Client and staff experiences of a co-located service for hepatitis C care in opioid substitution treatment settings in New South Wales, Australia. Drug Alcohol Depend. 2013;133(2):529–34. 23932843 10.1016/j.drugalcdep.2013.07.023

[47] Dawe J, Wilkinson AL, Asselin J, Carter A, Pedrana A, Traeger MW et al. Hepatitis C antibody testing among opioid agonist therapy recipients, Victoria, Australia, 2012 to 2020. Int J Drug Policy [Internet]. 2022;104:103696. https://www.sciencedirect.com/science/article/pii/S0955395922001153 10.1016/j.drugpo.2022.103696 35490624

[48] Marx R, Kahn JG. A narrative review of slow Medicine outcomes. J Am Board Fam Med. 2021;34(6):1249–64. 34772782 10.3122/jabfm.2021.06.210137

